# Understanding green supply chain information integration on supply chain process ambidexterity: The mediator of dynamic ability and the moderator of leaders’ networking ability

**DOI:** 10.3389/fpsyg.2022.1088077

**Published:** 2022-12-08

**Authors:** Tu Lyu, Yulin Guo, Huan Lin

**Affiliations:** ^1^School of Business, Qingdao University, Qingdao, China; ^2^School of Economics and Management, Dalian University of Technology, Dalian, China

**Keywords:** green supply chain, IT system integration, information sharing, supply chain dynamic capabilities, networking ability, supply chain process ambidexterity

## Abstract

Based on dynamic capability theory, this paper aims to explore the influence of green supply chain information integration (IT system integration and information sharing) on supply chain process ambidexterity (efficiency and flexibility) and expounds on the mediation mechanisms (supply chain dynamic capability) and the boundary condition (networking ability) between the two. Through the sample data test research model of 351 managers of manufacturing enterprises, it is found that information technology (IT) system integration can effectively promote the information sharing level of green supply chain enterprises. Supply chain dynamic capability partially mediates the influence of IT system integration and information sharing on supply chain process ambidexterity. Moreover, networking ability positively moderates the relationship between absorptive capacity, innovation capacity, and supply chain process ambidexterity, but does not play a significant role in the relationship between adaptive capacity and supply chain process ambidexterity.

## Introduction

Information technology (IT) drives the development of market informatization (Yu et al., 2021). Especially after the integration of emerging IT in the traditional manufacturing industry, it not only subverts the outmoded product manufacturing processes, but also brings more changes in new product types and manufacturing materials. In recent years, with the shortening of the consumption cycle and the rapid evolution of the consumer market, the manufacturing industry represented by electronic products has produced a large number of e-waste (Kamal et al., 2022). The environmental problems are mainly attributed to the fact that the environmental pollution caused by the product manufacturing and production process is deteriorating at a rate far faster than the rate that can be improved by resource compensation and product recycling and reuse measures, which will lead to the destruction of ecological environment and the depletion of natural resources in the long run (da Silva et al., 2021). The manufacturing industry is faced with the serious reality of environmental pollution and resource depletion, thus, the original resource-consuming production mode of enterprises will no longer be sustained (Assumpção et al., 2022). Only by grasping the balance point between environmental and economic performance can we maximize the economic benefits of enterprises (Zaid et al., 2018). In circular economy, the Green Supply Chain Management (GSCM) provides the resource optimization and it is seen as a solution to solve environmental problems and consumption patterns within the whole supply chain (Kazancoglu et al., 2018), which is a feasible way to balance the financial benefit and environmental protection in the manufacturing industry (Micheli et al., 2020). How to implement the green supply chain (GSC) effectively, to achieve the dual benefits of environmental and economic performance, is a critical issue that enterprises need to consider and solve.

The high performance of the GSCM, on the one hand, needs to pay attention to process efficiency to improve the efficient collaboration between enterprises in the GSC; on the other hand, it is necessary to pay attention to process flexibility (Bai et al., 2020; Bag and Rahman, 2021). As a process capability that can effectively cope with business changes and adjustments, process flexibility can coordinate the production tempo of processes and reduce the inertia of enterprises (Bai et al., 2020; Hou, 2020; Bag and Rahman, 2021). However, there is a contradiction between process efficiency and flexibility (Wadhwa et al., 2009; Zhu et al., 2020). Improving efficiency requires each process to enhance standardization and stability, reducing the process design’s flexibility; On the contrary, improving process flexibility requires a higher process adjustment range and lower standardization, thus reducing process efficiency (Wadhwa et al., 2009). To achieve high process efficiency and flexibility simultaneously, the research on supply chain process ambidexterity has become a hot topic in the academic field (Aslam et al., 2018; Pu et al., 2018).

Information integration is an essential basis to promote the successful operation of the GSC (Prajogo and Olhager, 2012; Wei et al., 2020; Lin, 2022), which can assist GSC enterprises in solving the dualism dilemma of process efficiency and flexibility. Different from the traditional manufacturing supply chain, the GSCM information integration mode particularly highlights the integration of resource consumption information, environmental damage information, and related policy information, and integrates and optimizes the information flow, logistics, and capital flow involved in the management process, to improve the operation efficiency of the entire supply chain (Lin, 2022). How to make the whole supply chain play a 1 + 1 > 2 multiplier effect through inter-organizational IT system integration and effective information exchange and sharing for enterprises in the supply chain has become the key to improving the overall performance of GSC (Ye and Wang, 2013; Hou, 2020). Supply chain information integration depends on the high degree of integration and penetration of information means and enterprise operation processes to show commercial value and improve internal management and reengineering business processes (Zhu et al., 2020). In the past, traditional enterprises usually regarded business process control and efficiency improvement as the core logic of all business process optimization (Aslam et al., 2018); however, when a large number of process models are integrated into the enterprise information management system, the information management system can, on the one hand, improve the management efficiency of the enterprises’ internal processes, but on the other hand, reduce the possibility and effect of process optimization due to process solidification, thus weakening the ability of internal processes to adapt to changes in the external dynamic environment. Mainly for enterprises in the GSC, the emergence of a large amount of information is testing the ability of enterprises to absorb and integrate information (Kong et al., 2021). How to complete information integration quickly and efficiently and share information with other enterprises in the supply chain simultaneously, to improve the overall information transmission efficiency of the GSC can significantly increase the efficiency of the GSC operation and the flexibility of the process.

At present, scholars are devoted to analyzing the problem of organizational ambidexterity (Cao et al., 2010; Katou et al., 2021), but few of them try to discuss the ambidexterity problem at the process level, with even less research focusing on the process ambidexterity in the GSC context. In addition, the current study has neglected to pay attention to the indirect path of information integration and supply chain process ambidexterity, and its internal transformation mechanism needs to be further explored. Scholars pointed out that core enterprises also need to rely on the dynamic capability to overcome the dilemma of core rigidity to maintain the relatively stable operation of supply chain processes (Nayak et al., 2020), while the resources, such as internal and external information, required by dynamic capability need to be acquired through supply chain information integration (Hou, 2020). Therefore, analyzing the relationship between information integration and GSC process ambidexterity is reasonable from the dynamic capability perspective. Based on dynamic capability, this study follows the research logic of information integration—dynamic capability—process ambidexterity, and focuses on analyzing the mediator role of supply chain dynamic capability in this path.

In addition, studies advocated exploring the mechanism of the performance output process from the perspective of leadership factors on resource integration (i.e., dynamic capability; von den Driesch et al., 2015; Murray et al., 2022). The networking ability of enterprise executives is also essential for the operation of the GSC network, which can help enterprise organizations to purposefully build, expand or modify their resource base, establish useful links through valuable upstream and downstream enterprises, and communicate with business partners in formal or informal forms to broaden social networks (Lyu et al., 2022), to strengthen collaboration and information interaction among GSC enterprises. This study believes that the leaders’ networking ability will expand the influence of dynamic capability on process ambidexterity. However, this mechanism of action has not been confirmed yet, which provides space for this study. Therefore, our study also tries to discuss and test the boundary condition of networking ability on organizational process ambidexterity.

Overall, this study explores the relationship between GSC information integration and supply chain process ambidexterity (process efficiency and flexibility) and the critical path to realizing process ambidexterity from information integration and information sharing. In this study, the dynamic capability is introduced as the mediator between information integration and process ambidexterity to explore the transformation mechanism of information integration. Networking ability is the moderator in the relationship between dynamic capability and process ambidexterity, thus revealing the boundary condition under which the dynamic capability plays a role. The study has made a theoretical contribution to the efficient operation of GSC. Firstly, it deconstructs the concept of information integration, and reveals the function path of information integration to promote GSC’s efficient operation and process flexibility. Secondly, it shows the mediating mechanism of dynamic capability in information integration to enhance supply chain process ambidexterity. Thirdly, it clarifies the boundary condition of supply chain dynamic capability to improve supply chain process ambidexterity, namely, the moderator of networking ability. By showing the critical path and mechanism of GSC to realize process ambidexterity, this study can provide theoretical support and practical countermeasures for implementing GSC practice.

## Literature review and theoretical basis

Referencing the main ideas of integrative review approach (Snyder, 2019), we reviewed the literature to identify the empirical evidence to assess, critique, and synthesize the literature and related concepts in the GSC field to sort out what we have known and develop new theoretical frameworks and perspectives of our research.

### Green supply chain management

The GSC is defined as “integrating environmental thinking into supply-chain management, including product design, material sourcing and selection, manufacturing processes, delivery of the final product to the consumers as well as end-of-life management of the product after its useful life” (Srivastava, 2007). The Green Supply Chain Management (GSCM) measures involve the whole life cycle of products, covering all stages of product design, production, packaging, and after-sales service (Tseng et al., 2019), which is a supply chain management mode adopted by enterprises to achieve sustainable development in an all-round way (Assumpção et al., 2022).

While pursuing economic benefits, the GSC emphasizes the compatibility between the activities, including planning, procurement, production, distribution and consumers, and environment (Abu Seman et al., 2019). Environment-friendly design, green procurement, internal environmental management, investment recovery strategy, and cooperation with upstream and downstream GSCs are considered five practical elements of the GSCM for manufacturing enterprises (Benzidia et al., 2021). Specifically, the environment-friendly design emphasizes that environmental protection should be fully considered during the product design (Fahimnia et al., 2015). Green procurement refers to assessing the influence of environmental factors in the supply chain during procurement to facilitate the recycling and reuse of resources and materials (Tseng et al., 2019). Internal environmental management refers to the management measures implemented by a single enterprise in its interior, which requires less cooperation from other enterprises in the supply chain (Tseng et al., 2019). The investment recovery strategy converts surplus materials and products into enterprise profits by selling surplus materials, reducing storage space, and recycling idle assets (Sarkis, 2012). The cooperation with upstream and downstream GSCs mainly includes collaboration with suppliers and customers in social and environmentally sustainable development (Mojumder and Singh, 2021).

Green supply chain management is considered to be a source of competitive advantage for companies (Yang et al., 2013; Benzidia et al., 2021). Yang et al. (2013) also found that the GSCM can improve enterprise and environmental performance. Zaid et al. (2018) further pointed out that the GSCM strategy can reduce production costs and protect the natural environment. Abu Seman et al. (2019) suggested that the GSCM considers environmental factors in product design, reduces the scarcity of enterprise resources, and assists enterprises in reducing environmental governance costs and business risks, so it is a critical way to improve enterprise performance. The GSCM building a green and environment-friendly corporate culture, saving energy, reducing waste generation, recycling waste products and materials, etc., thus focusing on the negative impact of business activities on the environment, which is conducive to improving the environmental performance of enterprises (Benzidia et al., 2021). In addition, under the background of increasingly homogeneous enterprises, differentiation is a crucial way to establish the green image of enterprises. As an effective embodiment of differentiation, the GSCM practices also help enterprises to build a green corporate image, and increase customer viscosity (Mojumder and Singh, 2021; Assumpção et al., 2022).

There is an irreconcilable contradiction between process efficiency and flexibility. High efficiency often means process solidification and lack of flexibility (Wadhwa et al., 2009; Zhu et al., 2020). Especially in the environment of GSC operation, an efficient collaboration of GSC enterprises requires specific solidified processes and standardized, formal operation modes, such as emphasizing the close cooperation between enterprises and some green energy and recycling enterprises, however, leading to the loss of vitality of the supply chain and the lack of dynamic environmental adaptability (Bag and Rahman, 2021). Organizations may face difficulties reshaping the supply chain when an individual green energy enterprise leaves the supply chain or a new enterprise is added to the chain. However, flexibility can make up for the changes in supply chain structure and process brought about by the dynamic environment, so that enterprises can maintain dynamic relationship maintenance and maintain the continuous operation of the chain through contingency (Bai et al., 2020). According to the ambidexterity theory, successful organizations simultaneously pursue two contradictory management objectives and strike a balance between them. The theory helps business executives break away from the traditional dualistic dilemma and creatively seek a compatibility solution (Fourné et al., 2019); that is, efficiency and flexibility are no longer “either/or” opposing concepts, but a pair of core capabilities that promote and support each other (Aslam et al., 2018; Pu et al., 2018). This paper defines process ambidexterity as the pursuit of high efficiency and flexibility during business processes. Process efficiency emphasizes improving existing products, processes, or capabilities of enterprises, resulting in reducing production costs and making the most effective use of existing resources simultaneously. Process flexibility is reflected in the adaptive production capacity during the production process, so the processing system adaptively responds to the environment’s uncertainty to meet customers’ diversified individual needs. The existing literature lacks a discussion on how to achieve process ambidexterity in the context of the GSCM.

### Supply chain information integration

In today’s competitive market environment, it is of great importance for core enterprise and its supply chain to timely transmit and process the information needed for supply chain decision-making, and improve and enhance the overall operational efficiency of the supply chain (Hou, 2020; Lin, 2022). Supply chain information integration is a means for enterprises to realize the sharing of intangible information in the supply chain network through cross-organizational information system integration, to speed up the exchange speed and frequency of information flow, logistics, and capital flow between core and upstream and downstream enterprises. It includes two main aspects: technical aspect (IT system integration) and social aspect (information sharing; Prajogo and Olhager, 2012). IT system integration, also known as cross-organizational electronic integration, refers to the vertical integration of the management system, operation system, manufacturing system, and infrastructure achieved by upstream and downstream enterprises in the supply chain through the configuration of specialized computer and communication systems (Wang et al., 2006). Supply chain information sharing is an information transmission method adopted by node enterprises in the supply chain during specific business activities or cooperation processes (Ye and Wang, 2013; Kong et al., 2021). This study holds that the two aspects of information integration coexist, and neither can be absent. Ignoring the role of IT systems cannot effectively deal with the intangible information between a core enterprise and its distribution network. However, excessive reliance on IT systems will lead to the homogenization tendency of various information system functions in enterprises, making it impossible for enterprises to communicate effectively and timely among supply chain members by building a tangible network. Only by simultaneously building technical capability and communication capabilities can those enterprises maximize the benefits during the overall operation of the supply chain. Pu et al. (2018) suggested that it can effectively improve supply chain ambidexterity by implementing E-logistic in the supply chain. However, it is unclear whether technology integration and information sharing in the GSC environment are helpful and how they function with the supply chain process ambidexterity.

### Supply chain dynamic capability

Dynamic capability building has become essential to developing and upgrading GSC (Song and Choi, 2018; Ye and Lau, 2022). Supply chain dynamic capability, regarded as the change capability of the supply chain, is the concrete embodiment of dynamic capability in a supply chain organization (Zhou and Li, 2010). It is not a simple addition of some conventions and procedures, but an adaptive capability formed by the lower-order capabilities of different members in the supply chain after coordination and continuous reconstruction (Beske et al., 2014; Hong et al., 2018), so that it can be the organization’s higher-order capabilities. Ye and Lau (2022) pointed out that the supply chain, as a complex system, needs to build dynamic capability to assist actors on the chain in dealing with various dilemmas and complex relationships positively and appropriately. Based on this point, the GSC dynamic capability can be considered as that in a dynamic environment, the actors on the chain can quickly respond to environmental changes and make optimal green strategic decisions of enterprises, to obtain sustainable competitive advantages by actively sensing and responding to changes in the external environment and adjusting, restructuring, optimizing and innovating existing resources, procedures and capabilities in the system.

Scholars gradually try to define the concept of supply chain dynamic capability from different perspectives. Defee and Fugate (2010) believed that the supply chain dynamic capability is the ability of participating members in the supply chain network to adjust the overall structure together to create new capabilities or improve old capabilities. Based on the process perspective of capability development, Nakano et al. (2013) regarded supply chain dynamic capability as a series of processes in which supply chain enterprises perceive, acquire, integrate, and reconstruct resources to adapt to market environment changes. This study focuses on the whole process of supply chain capability formation and reconstruction, as well as the integrity and systematization of the supply chain network. We defined it as the ability to adapt to environmental changes and maintain the relative stability of the whole network by absorbing and internalizing external resources, flexibly adjusting resource structure configuration, and optimizing and innovating business processes. Meanwhile, learning from the research of Wang and Ahmed (2007), the supply chain dynamic capacity is divided into three dimensions: absorptive capacity, adaptive capacity, and innovative capacity. The absorptive capacity refers to the ability of upstream and downstream enterprises in the supply chain to absorb valuable external information and turn it into usable information while identifying and acquiring it. The adaptive capacity emphasizes the enterprise’s elasticity and strategic flexibility, reflecting its capacity to reallocate resources and adjust its behavior to match the changing environment quickly. Innovation capacity refers to the ability of enterprises to continuously adapt their innovation strategic positioning and develop new processes or new products (services) through innovation behaviors and processes.

## Research model and proposed hypotheses

Social network theory holds that enterprises acquire resources in the external environment through network relationships, and bring benefits to both sides of the connection through effective communication of information and resources (Yamin and Kurt, 2018). The premise of stable operation of the supply chain process is to ensure real-time transmission of multi-party information related to operation (Şahin and Topal, 2019), and smooth and effective transmission of information depends on efficient information systems of enterprises, so that the support of IT infrastructure lays a foundation for the improvement of efficiency and flexibility of supply chain process (Pu et al., 2018). However, for the realization of ambidexterity, IT is far from enough to rely only on the support of IT infrastructure. Only by simultaneously building technical and social resources can those enterprises see the maximum benefits of operation (Prajogo and Olhager, 2012). Therefore, this study, with supply chain information integration as the antecedent variable, discusses the specific realization mechanism of GSC process ambidexterity from the aspects of IT system integration and information sharing among enterprises.

Dynamic capability theory emphasizes that in a dynamic environment, enterprises can quickly adapt to development needs by actively sensing and responding to changes in the external environment and adjusting, restructuring, optimizing, and innovating existing resources, procedures, and capabilities in the system, thus obtaining sustained competitive advantages (Beske et al., 2014; Hong et al., 2018). Information integration provides the necessary multi-party information for enterprise process operation, so it can be used as the condition of resources required to realize process ambidexterity (Wei et al., 2020). To make the process more stable to adapt to the changes in the complex environment, the process efficiency and flexibility tend to be more dynamic balance. It is also necessary to rely on the supply chain dynamic capability to better coordinate the external resource environment and internal management to achieve the smooth operation of the process (Aslam et al., 2018). Therefore, the supply chain dynamic capability (including supply chain absorptive capacity, supply chain adaptive capacity, and supply chain innovation capacity) are included in the research framework.

From the supply chain management perspective, social network theory also emphasizes organizational leaders’ positive role in maintaining and constructing a supply chain network (Falcone et al., 2022). Corporate leaders, such as CEOs, play a vital role in firm strategy because they are deft at developing supply chain plasticity through social connections (Abernethy et al., 2019). Many studies believed that senior managers, as essential leaders of enterprises, master the internal and external environmental information required for constructing the process ambidexterity (Fernández-Pérez et al., 2016), and are more able to control the operation process of enterprises freely, so that they can play a crucial role of “internal facilitator” during the process ambidexterity implementation. In other words, the networking ability of senior managers may play a moderating role in the path of realizing supply chain process ambidexterity.

**Figure 1:**
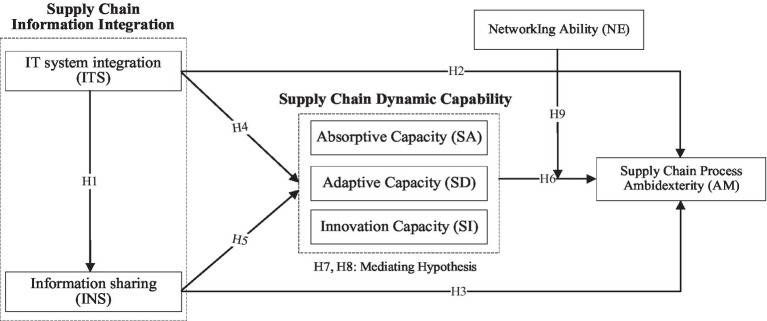
Research model.

Based on the above analysis, this study integrates the core views of social network theory and dynamic capability theory to construct a path relationship model among information integration, dynamic capability, and process ambidexterity in the GSC context. In addition, networking ability is introduced as a boundary condition to explore whether it plays a moderator role between dynamic capability and process ambidexterity. The research model is shown in Figure 1.

### IT system integration and information sharing

Information technology system integration refers to an integration of various internal information systems realized by enterprises by unifying the compatibility of data types and semantics, communication technologies, and software programs, connecting different software applications running on different hardware platforms, to realize the exchange of data and organizational processes (Wang et al., 2006). Cross-organizational IT integration enables enterprises with the information distributed in the GSC to transmit real-time product and service information through programs and functional modules related to the GSCM system (Lin, 2022). The emergence of inter-organizational information management systems, such as ERP and EDI, has gradually transformed the information transmission channel from a single linear structure to a cross-network chain structure, accelerated and expanded the flow and transmission of information between upstream and downstream enterprises in different directions, and promoted real-time information sharing among all participants in the whole supply chain process (Chen et al., 2019). The following hypothesis is proposed:

*H1*: IT system integration has a significant positive impact on information sharing among GSC enterprises.

### Information integration and process ambidexterity

Cross-organization information system has been widely used among GSC enterprises and has gradually become the basis of supply chain process operation. The highly integrated inter-organizational information system can support the integration of supply and demand and rapid data exchange, ensuring smooth process operation and thus gaining more market power than other supply chain members (Radhakrishnan et al., 2018; Lin, 2022). On the one hand, enterprises at each node of the supply chain can use inter-organizational information system integration to expand their information flow in the supply chain, which can help them reduce unnecessary process activities and replace inventory with information, thereby reducing the costs of manufacturing and various operational activities and improving process efficiency (Radhakrishnan et al., 2018). On the other hand, the integration of organizational IT infrastructure enhances the integration capacity of supply chain information flow and the coordination capacity among organizations, making the business process system more standardized, so that it can quickly combine diverse resources to meet different business needs, to improve process efficiency with process flexibility (Turker, 2014).

The frequency and quality of information sharing between core enterprises and their upstream and downstream enterprises embedded in GSC cannot be ignored (Prajogo and Olhager, 2012). On the one hand, to better improve the efficiency of the supply chain process through collaboration, all supply chain parties actively expand the scope and types of information shared, so that enterprise managers can more timely obtain and understand all kinds of information in production and operation, so that existing information can be used most effectively, thus improving the efficiency of process operation (Panahifar et al., 2018; Şahin and Topal, 2019). On the other hand, supply chain partners share information to ensure the consistency of business forecasting and scheduling between core enterprises and suppliers (Kong et al., 2021), so that the back-end can timely understand the resource collaboration of the front-end, so that the entire supply chain can quickly respond to changing market demands, thus further improving the flexibility of the supply chain. The following hypotheses are proposed:

*H2*: IT system integration has a significant positive impact on supply chain process ambidexterity.

*H3*: Information sharing has a significant positive impact on supply chain process ambidexterity.

### Information integration and dynamic capability

Information resources cannot directly bring sustainable competitive advantages to enterprises, only when they are further integrated and reconstructed to become valuable and inimitable resources can they play a more significant role (Mikalef and Pateli, 2017). However, dynamic capability focuses on the dynamic matching between business processes and environments, while emphasizing the reconstruction of internal and external resources, skills, and capabilities of an organization, so as to keep the process stability previously maintained by enterprises from being eroded (Song and Choi, 2018; Ye and Lau, 2022). Therefore, core enterprises need to rely on the dynamic capability to adapt to external market changes while keeping the supply chain relatively stable, and the internal and external information resources required by supply chain dynamic capability need to be acquired through supply chain information integration. In the following, the role of information integration on supply chain dynamic capability is further elaborated in three dimensions.

Cross-organization information system integration supports internal and external information resources for building supply chain dynamic capability. Firstly, procedures and functional modules related to supply chain management system can ensure the rapid and effective exchange and dissemination of data and information between organizations in the supply chain network, reduce information distortion and information asymmetry, and provide sufficient and accurate information resources for improving the adaptive capacity of the supply chain (Turker, 2014). Secondly, the cross-organization information system can enhance the interoperability between organizations through the configuration of specialized computers and communication systems, and improve the efficiency of information transmission and transfer, so that it is conducive to the dynamic adjustment of organizations to adapt to the external environment (Radhakrishnan et al., 2018). Finally, because the manufacturing system is highly technical and challenging to understand, hindering the further production and operation of the process to a certain extent, the production and manufacturing system can only be further integrated with the operating system and management system, so that the relevant data can be filtered and optimized for managers to understand and master the specific information of manufacturing sites (Mikalef and Pateli, 2017), thereby promoting a series of innovation activities in the production and manufacturing links, and improving the innovation capacity of supply chain enterprises.

Information sharing among supply chain enterprises can promote enterprises to better use of valuable information resources inside and outside the organization (Şahin and Topal, 2019), thus improving their dynamic capabilities. First of all, enterprises with higher absorptive capacity can absorb and internalize more relevant information available to them while acquiring information (Gölgeci and Kuivalainen, 2020), which benefits from the information sharing mechanism of the organization. Supply chain information sharing enables intangible information between distribution networks to be integrated and transferred among enterprises at each node (Kong et al., 2021), providing an effective way for the accumulation and creation of information at the management, operation, and strategy levels. Therefore, the continuous exchange of information is crucial to improving the organization’s ability to absorb external information. Secondly, the flexibility embodied in adaptive capacity comes from an organization’s understanding of the external environment, including technology, market, policy, and other relevant information (Oke et al., 2022). Therefore, the information shared by upstream and downstream enterprises in the supply chain, such as changes in external customer demands and technology/equipment changes, has an essential impact on the adaptive capacity. Raza et al. (2020) pointed out that the resource base, such as organizational information, is regarded as a trigger for adaptive behavior, and the core enterprises and upstream and downstream enterprises share the acquired information, such as orders, inventory, and recycling in real time to ensure synchronization with market changes, which is conducive to continuous dynamic adjustment of the supply chain structure system to adapt to changes in the external environment. Finally, innovation capacity emphasizes external flexibility, and pays more attention to coping with environmental changes through new product development or technological innovation. Panahifar et al. (2018) found that information sharing and interaction between core enterprises and node enterprises can provide enterprises with access to information, such as external technology and customer needs, add new and valuable content to existing organizational practices and production processes, or even completely rebuild existing infrastructure, thereby improving enterprises’ capacity to develop new processes or innovative products. Based on the above analysis, the following hypotheses are proposed:

*H4*: IT system integration has a significant positive impact on supply chain dynamic capability (a. absorptive capacity; b. adaptive capacity; c. innovation capacity).

*H5*: Information sharing has a significant positive impact on supply chain dynamic capability (a. absorptive capacity; b. adaptive capacity; c. innovation capacity).

### Dynamic capability and process ambidexterity

According to the dynamic capability theory, the characteristics and value of resources can change in a dynamic environment. Hence, organizations need to have the core capability to make better use of the resources they own, and dynamically adjust and optimize the allocation of resource structure, thus obtaining and maintaining the competitive advantages of enterprises (Beske et al., 2014). The supply chain dynamic capability captures the sum of the dynamic capabilities of all upstream, downstream, and core enterprises (Hong et al., 2018). The dynamic capability of a single enterprise alone cannot ensure that the supply chain achieves ambidexterity. Additionally, enterprises need to configure the dynamic capability embedded in the process with their upstream and downstream enterprises to cope with changes in the market environment, thereby improving the efficiency and flexibility of the supply chain.

Firstly, absorptive capacity positively impacts supply chain ambidexterity (Rojo et al., 2018). On the one hand, obtaining relevant market knowledge, technology, and other information from business relation members, such as partners, customers, and suppliers, is often related to the situation of the supply chain, so their digestion and absorption can help enterprises to adjust product/service strategies, improve production processes, and optimize production management promptly (Gölgeci and Kuivalainen, 2020; Oke et al., 2022), thus reducing production costs and raw material consumption, improving input and output rates, and enhancing supply chain efficiency. On the other hand, supply chain enterprises with strong absorptive capacity can quickly identify, absorb, and internalize information complementary to themselves (Yang L. et al., 2020), so that upstream and downstream enterprises have a shared resource base, such as information, so as to enhance the willingness to communicate and cooperate and the consistency of goals based on resonating with market demand and business operation, and thus increase the flexibility of infrastructure, department cooperation and process operation (Rojo et al., 2018).

Secondly, adaptive capacity is a driving force for the supply chain to adapt to the environment, because it matches the environment by coordinating and integrating the scattered resources and capabilities and adjusting the organization of the supply chain, so as to improve the existing production, process or capacity and make the most effective use of the existing resources (Eckstein et al., 2015; Raza et al., 2020). In addition, a higher adaptive capacity can better realize the dynamic integration and configuration of various resource combinations within the organization, providing a huge opportunity to improve the supply chain structure, and fundamentally enabling the processing system to quickly adjust relevant production factors, including production processes, production loads, etc., thus effectively responding to the variability of demands in supply chain and the changes in the environment promptly (Yang L. et al., 2020).

Finally, supply chain efficiency plays a crucial role in improving speed and performance, developing efficient information networks, etc., all of which need to be supported by supply chain innovation capacity (Zimmermann et al., 2020). Supply chain innovation capacity can assist enterprises in continuously improving production and process, and working out new operation strategies through innovative behaviors and processes (von den Driesch et al., 2015), so that enterprises can efficiently meet customer needs to improve process efficiency, and achieve the goal of seamless interaction between supply and demand. In addition, the supply chain with solid innovation capacity can effectively improve the operation efficiency of the processing system, develop and create diversified and differentiated products and high-quality services, so as to adapt to the changes in the market environment, gain the support of customers and the market (Yang L. et al., 2020), and improve the supply chain flexibility. Based on the above analysis, the following hypothesis is proposed:

*H6*: Supply chain dynamic capability (*a*. absorptive capacity; *b*. adaptive capacity; *c*. innovation capacity) has a significant positive impact on supply chain process ambidexterity.

### The mediator of supply chain dynamic capability

The mediator of supply chain dynamic capability on supply chain information integration and process ambidexterity can be explained using organization capability hierarchy theory. According to this theory, the capabilities possessed by an organization include low-order capabilities (basic capabilities required for daily production and operation) and high-order capabilities (the capabilities to help enterprises quickly respond to changes in the external environment for obtaining sustained competitive advantages), both of which can transform into each other and jointly affect enterprise performance and output (von den Driesch et al., 2015). Specifically, information management capabilities, such as information integration, are used to support the daily operation of enterprises. Through supply chain information integration, enterprises can use the resources, including internal and external information, to build their low-order/basic capabilities. Through transformation mechanisms, such as integration and reorganization, they can form high-order capabilities that can adapt to the changes in the environment and maintain the stability of enterprise processes, that is, improve the supply chain dynamic capability (Mikalef and Pateli, 2017). Through the continuous expansion and integration of information resources, as well as the improvement and smooth operation of subsequent processes, information integration (low-order capabilities), and dynamic capability (high-order capabilities) are coordinated to achieve the best state. While improving the supply chain efficiency, it can also ensure the rational allocation of resources through business process optimization, design, and develop high-quality and high-standard products/services to improve the supply chain flexibility (Rojo et al., 2018; Yu et al., 2018). Therefore, this study believes that the influence of supply chain information integration on supply chain process ambivalence can be transmitted through supply chain dynamic capability. Based on the above analysis, the following hypotheses are proposed:

*H7*: IT system integration, mediated by supply chain dynamic capability (a. absorptive capacity; b. adaptive capacity; c. innovation capacity), has an indirect effect on supply chain process ambidexterity.

*H8*: Information sharing level, mediated by supply chain dynamic capability (a. absorptive capacity; b. adaptive capacity; c. innovation capacity), has an indirect effect on supply chain process ambidexterity.

### The moderator of networking ability

During the supply chain operation, enterprises cannot generate all the required resources internally, so they must obtain some resources needed from the outside to ensure the excellent operation of their supply chain. According to the social network theory, the social network of an enterprise is an essential source for obtaining market forecast information, customer demand changes and resources needed for production and operation (Yamin and Kurt, 2018), and heterogeneous resources absorbed in the social network provide necessary nutrients for the development of its supply chain. Networking ability refers to the ability of senior managers to expand social networks (Cao et al., 2010), and it can expand social networks by establishing effective connections with valuable upstream and downstream enterprises, and often communicating with business partners in formal or informal forms. Both sides promote the embedding of information beneficial to their own and their partners’ development during the interaction process.

Using leaders’ network relations positively impacts the transformation of the dynamic capability to business performance at the enterprise level (von den Driesch et al., 2015). Therefore, this study holds that the leaders’ networking ability can expand the impact of dynamic capability on process ambidexterity. First, the networking ability of senior managers constantly provides supply chain enterprises with the capability to discover, evaluate, and select suitable partners and establish close relationships (Ma et al., 2009). In the case of strong networking ability, intimate relationships can provide an enterprise with more information necessary for supply chain operation (Abernethy et al., 2019), such as competitors’ green strategies to provide a rich accumulation of information resources for improving absorptive capacity and enable the enterprise to acquire and accumulate new resources, thereby internalizing them into its advantages and strengthening the process efficiency and flexibility. Secondly, the stronger the networking ability of senior managers is, the more they can lead their affiliated enterprises to keep pace with market changes by capturing market data and identifying fundamental modes, so that they can better drive their enterprises to constantly restructure and allocate their resources to improve the adaptive capacity of the supply chain (Ma et al., 2009), thus enabling the organization to adapt to the changes in the external market environment and further improving the process ambidexterity. Thirdly, the social network constructed by leaders provides a wide range of information access and an effective path for screening and disseminating information (von den Driesch et al., 2015). The stronger the networking ability is the more valuable information for their development through screening information CEOs will obtain, to use it for the improvement and innovation of the supply chain operation methods. Therefore, they can continuously improve the positive effect of the supply chain innovation capacity on the supply chain efficiency and flexibility through innovative behaviors and processes (Fernández-Pérez et al., 2016). Based on the above analysis, the following hypothesis is proposed:

*H9*: Networking ability positively moderates the relationship between supply chain dynamic capability (a. absorptive capacity; b. adaptive capacity; c. innovation capacity) and supply chain process ambidexterity.

## Methodology

### Variable measurement

The measurement scale used in this study was derived from the literature to improve measurement accuracy. We made some modifications to the wording and expressions to fit our study scenario of GSC management. All the variables were measured on a five-point Likert scale, 1–5 represents the degree of acceptance of the subjects to the content described in the measurement items, in detail, 1 implied strongly disagree, and 5 implied strongly agree.

1. Supply chain information integration (II) scales were adapted from Prajogo and Olhager (2012) and Ye and Wang (2013), including two dimensions: IT system integration (ITS) and information sharing (INS). The ITS scale includes four items, such as “our enterprise can obtain the business data that is related to the upstream and downstream enterprises in the green supply chain through the cross-organizational information systems”; and the INS scale includes five items, like “our enterprise is more willing to share information, such as green energy, technology, and orders, with green supply chain partners.”
2. Supply chain dynamic capability (DC) scales were adapted from Wang and Ahmed (2007), Zhou and Li (2010), and Eckstein et al. (2015), including three dimensions: absorptive capacity (SA), adaptive capacity (SD), and innovation capacity (SI). SA scales include four items, such as “the green supply chain in which our enterprise is located has strong abilities to use newly acquired resources”; SD scales include four items, such as “our enterprises can adjust the product mix according to the market demands”; and SI scales include five items, such as “our enterprise’s green supply chain is improving its production practices much faster than competitors.”
3. Supply chain process ambidexterity (AM) in this study captures the pursuit of high efficiency and flexibility in the organizational processes. Thus, we use the product term of efficiency (SE) and flexibility (SF) to represent AM measurement, which reflects the interaction between supply chain efficiency and flexibility. The larger the absolute number of interaction values, the higher the degree of AM. SE scales include four items adapted from Zhu et al. (2020), such as “the suppliers in the green supply chain can quickly cooperate with our enterprise to design and produce new productions”; SF scales also include four items adapted from Zhu et al. (2020), such as “when there is a big change in customer demand, the suppliers in the green supply chain can cooperate with us to make a quick response.”
4. Networking ability (NE) scales were adapted from Cao et al. (2010), including four items, for example, “CEO in our enterprise often communicates with other enterprise leaders in the green supply chain,” “CEO in our enterprise is good at connecting with valuable enterprises in the green supply chain.”

### Pilot study

This study selects manufacturing enterprises with green manufacturing experience to conduct a small sample pilot study, and the survey objects are middle and senior managers who are relatively familiar with the enterprise. A total of 97 questionnaires were collected, and 89 valid questionnaires were obtained by eliminating incomplete and extremely regular questionnaires. This pilot study serves two purposes. Firstly, to ensure content validity, the respondents’ opinions on the accuracy, clarity, and conciseness of the questionnaire were investigated. Items with expression problems in the feedback were adjusted. Secondly, the reliability and validity of the sample data were tested. Results showed that the Cronbach’s *ɑ* coefficient and composite reliability (CR) of each variable were all above 0.7, and average variances extracted (AVE) and factor loading were all above 0.5, indicating that the scale involved in this study could measure the relevant variables more accurately.

### Data collection

Green supply chain management adoption is still at an early stage in China. An outline of the Chinese government’s industrial green development plan (2016–2020) highlighted green manufacturing as a critical direction of the national industrial transformation (China Government News, 2016). The report to the 20th CPC National Congress also underscored that China would continue to pursue “green and sustainable” industrial transformation and development direction. Under this scenario of the GSC development in China, our study focuses on one of the most polluting manufacturing industries in China, the electronics industry, to explore the relationship between information integration and process ambidexterity.

Our study used the electronic survey method to collect data. Considering the restrictions of objective factors such as financial support and time and the convenience of issuing and recycling questionnaires, we selected electronic manufacturing enterprises in Yantai and Qingdao in China to conduct the survey. CEOs in these manufacturing enterprises, and the head of departments in manufacturing, purchasing, logistics, recycling, and other operation departments are selected as the survey objects to complete the questionnaires. The reason is that these middle and senior managers are familiar with the internal and external operation processes of the enterprise, and have a deep understanding of the enterprises’ green strategy and GSC operation than ordinary employees. The electronic questionnaire was sent *via* email to the subjects. They were invited to return the completed questionnaire within the specified time limit (within 2 weeks of sending out our request email). The overdue questionnaires will be treated as invalid. We got 498 survey samples after the collection process. After removing the invalid questionnaires with incomplete filling and extreme regularity, 351 valid samples were finally obtained, with an overall effective rate of 70.5%. Table 1 shows the characteristics of enterprise samples.

**Table 1:** Sample characteristics (*N* = 351).

Category		Number	%
Enterprise Size	Less than 50 people	20	5.69
	51–200 people	111	31.62
	201–500 people	97	27.64
	501–1,000 people	79	22.51
	More than 1,000 people	44	12.54
Enterprise Years	Less than 2 years	89	25.36
	2–5 years	114	32.48
	5–8 years	78	22.22
	8–10 years	47	13.39
	More than 10 years	23	6.55
Industries	Manufacturing of communication equipment, computers, and other electronic equipment	72	20.51
	Electronic instruments, meters, equipment manufacturing (computer communication equipment, electronic devices, etc.)	78	22.22
	Electronic component manufacturing (resistors, inductors, printed circuit boards, etc.)	55	15.67
	Electrical device manufacturing (vacuum tube, integrated circuit, etc.)	46	13.11
	Manufacturing special materials (semiconductor materials, high-frequency magnetic materials, etc.)	54	13.38
	Other electronics-related manufacturing	46	13.11

## Data analysis and results

### Common method bias and multicollinearity

Common method bias (CMB) and multicollinearity may jeopardize the structural equation modelling (SEM) results. First, we tested the CMB using the Harmon’s single-factor test method (Podsakoff et al., 2012). Principal component factor analysis showed that all factors explained 78.333% of the total variance. The variance interpretation rate of the first factor was 10.854%, less than 50%, indicating that there is no serious common method bias. We then used Pearson’s two-tail test as a correlation analysis to test the multicollinearity. As shown in Table 2, the correlation coefficient between variables was less than the threshold value of 0.75 (Blacconiere and Patten, 1994), which indicates that the multicollinearity problem was excluded.

**Table 2:** Correlation matrix.

Constructs	1	2	3	4	5	6	7	8
1. ITS	1							
2. INS	0.376^**^	1						
3. SA	0.443^**^	0.435^**^	1					
4. SD	0.567^**^	0.524^**^	0.549^**^	1				
5. SI	0.445^**^	0.332^**^	0.448^**^	0.462^**^	1			
6. SE	0.469^**^	0.420^**^	0.509^**^	0.536^**^	0.441^**^	1		
7. SF	0.522^**^	0.444^**^	0.517^**^	0.550^**^	0.526^**^	0.507^**^	1	
8. NE	0.032	−0.021	0.132^*^	0.059	0.251^**^	0.030	0.074	1

^**^p < 0.01, ^*^p < 0.05; Pearson correlation coefficient adopts two-tail test.

### Measurement validation

We used statistical analysis software of SPSS 26.0 and MPLUS 8.6 to test the reliability and validity of the data. As shown in Table 3, the Cronbach’s alpha (CA) and composite reliability (CR) scores were higher than 0.7 specified by Bagozzi and Yi (1988), and scores of average variances extracted (AVE) were higher than the threshold of 0.5 (Bagozzi and Yi, 1988). Moreover, the range of factor loadings was 0.782–0.896, which was greater than the recommended reference value of 0.6 (Chin, 1998). Thus, the data had good reliability and convergent validity.

**Table 3:** Indicator reliability and convergent validity statistics.

Constructs	Items	Factor loadings	Mean	S.D.	AVE	CR	CA
ITS	ITS1	0.854	3.158	0.654	0.770	0.932	0.900
	ITS2	0.882					
	ITS3	0.888					
	ITS4	0.886					
INS	INS1	0.872	3.523	0.516	0.699	0.921	0.892
	INS2	0.880					
	INS3	0.856					
	INS4	0.782					
	INS5	0.785					
SA	SA1	0.896	3.956	0.603	0.744	0.935	0.912
	SA2	0.863					
	SA3	0.843					
	SA4	0.829					
	SA5	0.879					
SD	SD1	0.869	4.187	0.588	0.741	0.920	0.883
	SD2	0.850					
	SD3	0.843					
	SD4	0.881					
SI	SI1	0.786	4.176	0.528	0.661	0.907	0.871
	SI2	0.824					
	SI3	0.819					
	SI4	0.807					
	SI5	0.827					
SE	SE1	0.871	4.377	0.552	0.741	0.920	0.883
	SE2	0.865					
	SE3	0.843					
	SE4	0.865					
SF	SF1	0.839	4.444	0.536	0.720	0.911	0.870
	SF2	0.873					
	SF3	0.862					
	SF4	0.818					
NE	NE1	0.879	4.058	0.664	0.780	0.934	0.905
	NE2	0.876					
	NE3	0.895					
	NE4	0.882					

The study used the competition model comparison method to test the discrimination validity and compare a series of factor models with nested relationships (Salekin et al., 2014). The benchmark model is composed of eight factors, including ITS, INS, SA, SD, SI, SE, SF, and NE, and the benchmark model was compared with the seven-factor, six-factor, five-factor, four-factor, three-factor, two-factor, and one-factor competition models. Table 4 indicates that the fitting effect of the eight-factor model on the actual data was better than that of other nested models, which showed that the eight-factor benchmark model was the most ideal. Thus, the constructs had good discriminant validity.

**Table 4:** Confirmatory factor analysis of variable discrimination validity.

Model	Factors	χ^2^	df	χ^2^/df	TLI	CFI	RMSEA	SRMR
Model 1 (Eight factors)	ITS, INS, SA, SD, SI, SE, SF, NE	992.473	532	1.866	0.937	0.944	0.050	0.039
Model 2 (Seven factors)	ITS+INS, SA, SD, SI, SE, SF, NE	1765.756	539	3.276	0.835	0.851	0.081	0.069
Model 3 (Six factors)	ITS, INS, SE, SF, NE, SA + SD + SI,	1999.028	545	3.668	0.807	0.823	0.087	0.069
Model 4 (Five factors)	ITS+INS, SE, SF, NE, SA + SD + SI,	2764.255	550	5.026	0.709	0.731	0.107	0.091
Model 5 (Four factors)	ITS+INS + SA + SD + SI, SE, SF, NE	3167.235	554	5.717	0.659	0.682	0.116	0.096
Model 6 (Three factors)	ITS+INS + SA + SD + SI, SE + SF, NE	3491.221	557	6.268	0.619	0.643	0.123	0.091
Model 7 (Two factors)	ITS+INS + SA + SD + SI + SE + SF, NE	3723.128	559	6.660	0.590	0.615	0.127	0.095
Model 8 (One factor)	ITS+INS + SA + SD + SI + SE + SF + NE	4609.762	560	8.232	0.477	0.507	0.144	0.116

“+” means that the front and rear factors are merged into one factor.

### Hypothesis testing

We tested the structural equation model through the software of MPLUS 8.6. As shown in Table 5, the path from ITS to INS was significant ( β =0.576, *p* < 0.001), thus H1 was supported. The paths from ITS ( β =0.299, *p* < 0.001) and INS ( β =0.150, *p* < 0.01) to AM were significant, thus H2 and H3 were supported. Moreover, the paths from ITS to SA ( β =0.498, *p* < 0.001), SD ( β =0.660, *p* < 0.001), and SI ( β =0.572, *p* < 0.001) were significant, and paths from INS to SA ( β =0.312, *p* < 0.001), SD ( β =0.362, *p* < 0.001), and SI ( β =0.191, *p* < 0.01) were also significant, supporting H4*a ~ c* and H5*a ~ c*. The paths from SA ( β =0.240, *p* < 0.001), SD ( β =0.210, *p* < 0.001), and SI ( β =0.220, *p* < 0.001) to AM were significant, thus H6*a ~ c* were supported.

**Table 5:** Significance analysis of the direct effects.

Path	Standardized path coefficients	S.E.	Significance
H1: ITS→INS	0.576^***^	0.090	Yes
H2: ITS→AM	0.299^***^	0.067	Yes
H3: INS → AM	0.150^**^	0.055	Yes
H4*a*: ITS→SA	0.498^***^	0.095	Yes
H4*b*: ITS→SD	0.660^***^	0.074	Yes
H4*c*: ITS→SI	0.572^***^	0.084	Yes
H5*a*: INS → SA	0.312^***^	0.061	Yes
H5*b*: INS → SD	0.362^***^	0.054	Yes
H5*c*: INS → SI	0.191^**^	0.058	Yes
H6*a*: SA → AM	0.240^***^	0.053	Yes
H6*b*: SD → AM	0.210^***^	0.052	Yes
H6*c*: SI → AM	0.220^***^	0.048	Yes

^**^p < 0.01, ^***^p < 0.001.

A bootstrap approach was adopted to evaluate the mediation effects proposed in H7a ~ c and H8a ~ c. The number of samples was set to 1,000, and the confidence level was set to 95%. Compared with the traditional methods such as causal stepwise regression and the Sobel test, this method can not only test the mediation effects exerted by all parallel mediation variables but also compare whether there were significant differences in the effects of different mediation paths, which was a more effective and reasonable method for testing the mediation effects (Preacher and Hayes, 2004). As shown in Table 6, the indirect effect of ITS on AM through SA was 0.078 (SE = 0.025, *p* < 0.01). The confidence interval of the indirect effect did not contain 0 at a 95% confidence interval (CI; Preacher and Hayes, 2004), indicating that the mediating effect of ITS between SA and AM was significant. Moreover, ITS had a significant direct effect on AM, with an effect of 0.196 (SE = 0.045, *p* < 0.001). Therefore, ITS partially mediates the effect between SA and AM. Similarly, SD and SI partially mediate the effect between ITS and AM, and the effect of INS and AM was partially mediated by SA, SD, and SI. Thus, H7a ~ c and H8a ~ c were supported.

**Table 6:** Mediating effect testing results.

Path	Estimate	SE	Bias-corrected 95% CI	P
			Lower	Upper	
H7*a*: ITS→SA → AM	0.078	0.025	0.037	0.135	0.002
H7*b*: ITS→SD → AM	0.090	0.025	0.047	0.148	0.000
H7*c*: ITS→SI → AM	0.082	0.024	0.042	0.137	0.001
H8*a*: INS → SA → AM	0.075	0.021	0.040	0.125	0.000
H8*b*: INS → SD → AM	0.076	0.022	0.039	0.124	0.001
H8*c*: INS → SI → AM	0.042	0.016	0.017	0.080	0.010

The latent moderated structural equation method was adopted to assess the moderation roles of NE between the three dimensions of supply chain dynamic capability (e.g., SA, SD, and SI) and AM. As shown in Table 7, the interactions between NE and SA ( b = 0.158, *p* < 0.05)/SI ( b =0.190, *p* < 0.001) had a positive and significant impact on AM, supporting H9a and H9c. However, the interaction between NE and SD had no significant impact on AM ( b = − 0.104, *p* > 0.05), and H9b was not supported.

**Table 7:** Moderating effect testing result.

Constructs	AM
SA	0.282^***^
SD	0.383^***^
SI	0.474^***^
NE	−0.149^*^
SA*NE	0.158^*^
SD*NE	−0.104
SI*NE	0.190^***^

^*^p < 0.05, ^***^p < 0.001.

**Figure 2:**
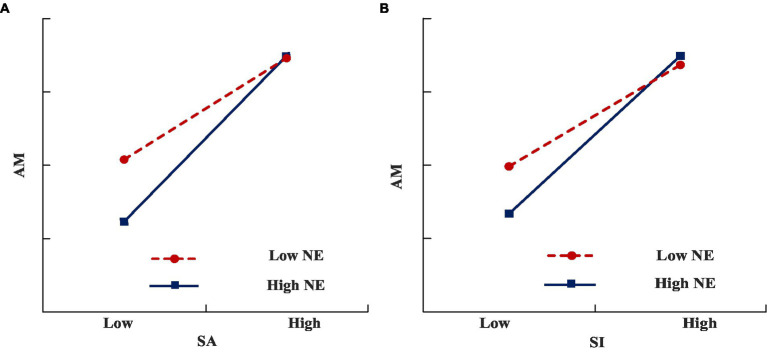
Moderating effect testing result (NE). **(A)** The moderating effect of NE between SA and AM. **(B)** The moderating effect of NE between SI and AM.

We adopted a method suggested by Aiken and West (1991) to further validate the moderating roles of NE. As shown in Figure 2A, the correlation between SA and AM became more significant when NE was higher than it was lower. Similarly, as shown in Figure 2B, the correlation between SI and AM became more significant when NE was higher than when it was lower. The positive moderation roles of NE were further confirmed.

## Discussion

### Key findings

With Chinese manufacturing enterprises as the research object, this paper empirically tests the influence of information integration among GSC enterprises on supply chain process ambidexterity, and explains the mechanism of action between them (supply chain dynamic capability) and the boundary conditions (networking ability). The research conclusions are as follows:

1. IT system integration can promote the information sharing level of supply chain enterprises, which is consistent with the research conclusion of Feng et al. (2020). The cross-organization information system provides platform support for information sharing among GSC enterprises, thus enabling the rapid and accurate exchange and sharing of data and information among organizations, and better implementing the interaction and cooperation among all members in the GSC. The study expands the existing research (Partanen et al., 2020) and verifies the positive effect of information integration on supply chain process ambidexterity from the technical and social aspects. Specifically, the information integration, including IT system integration (technical aspect) and information sharing (social aspect), can expand an enterprise’s information flow, promote the exchange of all kinds of information, and provide the necessary information and other resources for the supply chain to achieve high efficiency and flexibility during the business process. On the one hand, organizations can obtain all kinds of information about production and operation more quickly and accurately, and make the whole production and operation process more orderly by adjusting the operation mode, so as to speed up the process operation. On the other hand, high-level information integration can assist organizations in obtaining green demands and requirements promptly, and respond quickly to improve process flexibility.
2. Previous studies have confirmed the positive role of supply chain ambidexterity on green supply chain management (Khan et al., 2021); however, few studies have explored how to promote the ambidexterity of green supply chain. Our study tries to explore the mechanisms of information integration on supply chain ambidexterity, and found that the dimension of supply chain dynamic capability plays a partial mediator role in the influence of IT system integration and information sharing on supply chain process ambidexterity. The information integration among GSC enterprises is conducive to integrating the information and other resources of upstream and downstream enterprises, and realizing the process ambidexterity. However, their dynamic capabilities also need to support the integration and effective use of information. Information integration promotes frequent and close information interaction among the organization members on the chain, thereby enabling effective information transmission and sharing among various entities (Prajogo and Olhager, 2012; Feng et al., 2020). To understand and apply all kinds of information shared by enterprises in the supply chain, their dynamic capabilities need to be internalized into their own advantages. Precisely, by absorbing different types of information from upstream and downstream of the supply chain, they can adjust their resource allocation in real-time, to propose more complete and creative operation methods and improve the overall competitiveness of the GSC. This transformation mechanism can digest the information resources obtained by information integration through internal dynamic capability, and then produce the results conducive to the realization of supply chain process ambidexterity.
3. Previous studies using networking ability as the moderator to understand how supply chain ambidexterity worked on performance (Partanen et al., 2020; Khan et al., 2021), while, our study involved the networking ability to capture the generation mechanisms of supply chain ambidexterity, i.e., how dynamic capabilities worked on supply chain ambidexterity under the condition of networking ability. Results confirmed that networking ability positively moderates the relationship between the supply chain absorptive capacity, innovation capacity, and process ambidexterity, but the positive moderating effect of networking ability on supply chain adaptive capacity and process ambidexterity has not been verified. No significant moderator effect may be due to many situational variables that affect the relationship between adaptive capacity and supply chain process performance, such as homogeneity between enterprises and suppliers, between users and competitors (Schoenherr and Swink, 2015), and product complexity (Eckstein et al., 2015). Therefore, even if leaders’ high-order networking ability contributes to promoting the improvement of supply chain process efficiency and flexibility, the effect of internal and external market environment or other situational factors faced by enterprises may be more obvious, thus greatly weakening the moderator role of networking ability in this path.

### Theoretical contributions

Green supply chain management can gain environmental benefits by integrating circular economy principles (Genovese et al., 2017); our study provided empirical evidences to promote the upgrading of GSC in the manufacturing industries and the development of circular economy at the industrial level.

Firstly, in the GSCM scenario of the manufacturing industry, from the perspective of supply chain information integration, the relationship between it and supply chain process ambidexterity is further discussed. The researches on information integration among enterprises in the supply chain focus on the technical level (Zeng and Pathak, 2003; Yang R. et al., 2020), and it is rare to use empirical analysis methods to explore information integration issues in GSC strategic management. In addition, in the limited empirical research on information integration among enterprises in the supply chain, scholars only focus on the importance of IT system integration (Khuntia et al., 2021) or information sharing (Partanen et al., 2020) as a means of information integration. This study focuses on the core concept of supply chain information integration. It analyzes it from the two dimensions of technology and society, namely IT system integration and information sharing, to make up for the defects of previous studies that focus too much on a single perspective, and build the overall conceptual framework of supply chain information integration in a more systematical and complete manner. Meanwhile, by building the research model of improving supply chain process performance through information integration based on the ambidexterity theory, this study can broaden the application scope of ambidexterity and further enrich the empirical research on the relationship between information integration and process ambidexterity.

Secondly, it can reveal the mediator mechanism of information integration to improve the GSC process ambidexterity, namely, the mediator of supply chain dynamic capability. Some scholars have discussed the influence of information integration on supply chain performance from the perspectives of logistics integration (Prajogo and Olhager, 2012; Prajogo et al., 2016) and social exchange (Wu et al., 2014), but few scholars can incorporate dynamic capability into this framework. Therefore, based on the research of Prajogo and Olhager (2012), this study introduced supply chain dynamic capability as a mediator variable into the research on the relationship between information integration and process ambidexterity, built a relationship integration model including information integration, dynamic capability, and supply chain process ambidexterity, and then deeply discussed the path and mechanism of information integration to further realize supply chain process ambidexterity by promoting dynamic capability. The research model in this paper is an important supplement to the relationship between upstream and downstream information integration and supply chain process ambidexterity, and also makes a reasonable path derivation for the antecedents and consequences of supply chain dynamic capability. This study can make up for the lack of attention on process ambidexterity in existing GSCM research (Khan et al., 2021), reveal the information integration driving mechanism to achieve process ambidexterity, and enrich the GSCM literature.

Thirdly, it can clarify the boundary conditions of supply chain dynamic capability to improve supply chain process ambidexterity, namely, the moderator of networking ability. Liu et al. (2021) pointed out that exploring the process mechanism of performance output from both individual and organizational levels is more consistent with the enterprise core competence transition process. Therefore, exploring the realization mechanism of supply chain process ambidexterity is reasonable from the perspectives of leadership factors and dynamic capability. However, most of the existing studies separated the relationship between leadership factors and dynamic capability, without discussing the impact of their interaction on enterprise output (Najmi et al., 2018). Therefore, this study introduces networking ability as a moderator variable to explore the impact of its interaction with dynamic capability on supply chain process ambidexterity, and further extends the analysis framework of supply chain information integration—supply chain dynamic capability—supply chain process ambidexterity. The study can further reveal the role of senior enterprise leaders in the generation of GSC process ambidexterity, and expand the explanation scope of competency hierarchy theory.

### Practical implications

This paper probes into the influencing factors of high efficiency and flexibility of the supply chain from multiple perspectives, and provides a reference for supply chain managers to realize the supply chain process ambidexterity. The practical implications can be listed as follows:

Firstly, we should pay attention to IT infrastructure construction, and strengthen the willingness for information sharing among GSC enterprises. It can be found from the research conclusions that high process efficiency and high flexibility are not a pair of inextricable cruxes, so that enterprises should soberly abandon the traditional thinking of “trade-offs” and “either/or,” and try to use the dual review of “this and that” to have it both ways of solution. Moreover, information integration is one of the leverage solutions to resolve this problem. With the help of IT integration and supply chain enterprise information sharing, on the one hand, it is necessary to ensure a good match between the IT integration system and GSC processes. On the other hand, it is also essential to pay attention to the use of IT integration to provide platform support for information sharing between supply chain enterprises, thus providing real-time and accurate upstream and downstream information for process operation, and achieving process ambidexterity. Therefore, enterprises need to continue to improve their IT infrastructures, such as enterprise application software and information integration systems, to provide sharing platform support for information transmission and sharing among node enterprises in the supply chain. Node enterprises should strengthen the communication and interaction of their cultures and strategies, and establish the common green goal and vision of all enterprises and employees in the supply chain, to improve their willingness to share information and improve the efficiency and flexibility of the supply chain to a greater extent.

Secondly, we should cultivate and construct the supply chain dynamic capability and coordinate the balance of sub-capabilities. This study found that dynamic capability can provide operational power for information resources to realize the supply chain process ambidexterity through absorption, integration, adaptation, reconstruction, and processing innovation. Therefore, the construction of dynamic capability can further provide a new way to obtain both efficiency and flexibility of GSC. Supply chain dynamic capability integrates the dynamic capabilities of upstream and downstream enterprises, including core enterprises, so that it is the sum of the dynamic capabilities of all enterprises and a series of sub-capabilities of each enterprise. Therefore, enterprises should not only strive to cultivate their own dynamic capabilities, but also make them innovative by absorbing multi-party information, optimizing, and adjusting the supply chain structure configuration, and improving the operation methods. In addition, considering the differences in the capacity, and demand factors of each sub-dimension, enterprises should also balance the relationship between their dynamic capabilities and each sub-dimension, and work together to ensure the improvement of process ambidexterity.

Thirdly, we should create a suitable environment for resource interaction and give play to the moderate initiative of enterprises. In the research on how existing resources affect business performance, either the impact of environmental factors on enterprise performance was ignored, holding that enterprise’s own resources and capabilities should be regarded as the basis for improving business capabilities, or they paid too much attention to the process of acquiring relational resources while ignoring the dynamic role of enterprises themselves. Therefore, each node enterprise in the supply chain should not only strengthen its internal function, namely, the improvement of dynamic capability, but also attach great importance to information communication and resource interaction with related enterprises. More social support and financial resources should be gathered by improving the frequency of contact between upstream and downstream enterprises in the supply chain. Only by taking overall consideration, maintaining a good partnership between upstream and downstream enterprises, and meanwhile absorbing, adjusting, and transforming the acquired relation resources based on enterprise initiative, can the influence of the interaction between the two on process ambidexterity be brought into play to a greater extent.

Fourthly, we should cultivate the networking ability of senior managers and promote the transformation of dynamic capability into supply chain process ambidexterity. The networking ability of senior managers is of great significance for GSC operation and strengthening the coordination among supply chain members. Senior managers should participate in the construction and operation of GSC as liaisons and coordinators, strengthen the discovery and interaction of resources among member enterprises, and promote the absorption and innovation of resources and the cultivation and transformation of enterprise dynamic capability. It is necessary for senior managers to have keen network insight and construction capacity, identify the core resources of GSC enterprises, complete resource allocation through relationship coordination, build a common vision of the network, and improve process performance and flexibility.

### Research limitations and prospects

This study has the following limitations to be further explored in the future. Firstly, the cross-sectional data were collected by questionnaire method to verify the model. In fact, the information integration among enterprises in each node of the supply chain and the dynamic capability of the supply chain can have the characteristics of dynamic changes over time. In the future, longitudinal data research can be considered to have a more accurate understanding of the evolution rules of inter-enterprise information integration and dynamic capabilities. Secondly, according to the contingency theory, the interaction between variables requires specific situational conditions. The effect of networking ability was only considered in this paper, but the influence of supply chain information integration and dynamic capability on supply chain process ambidexterity may be affected by many other organizational and situational factors (such as market/technology uncertainty, organizational structure, and organizational culture). Therefore, more moderator variables will be considered in future research, in order to have a more comprehensive understanding of the boundary conditions of information integration and dynamic capability affecting supply chain process ambidexterity. Thirdly, as for the realization mechanism of supply chain process ambidexterity, its influencing factors mainly include information integration, dynamic capability, and networking ability among supply chain enterprises. However, the empirical analysis method used in this paper ignores the complex influence of the interaction of these factors, which will be further discussed by the qualitative comparative analysis (QCA) of fuzzy sets in the future. Finally, this study takes China’s electronic manufacturing industry as an example to test our research model. In the future, more in-depth discussions can be carried out on the implementation scenarios of GSC of other manufacturing enterprises to expand the multi-scenario applicability of the model.

## Conclusion

This study discusses the impact of GSC information integration on process ambidexterity by introducing dynamic capability as the mediator and leaders’ networking ability as the moderator, which is an expansion and supplement to the research on GSCM of manufacturing enterprises. Our research results verify the mediation effect of dynamic capability between information integration and supply chain process ambidexterity, and the different moderating effects of networking ability between different dimensions of dynamic capability and process ambidexterity. The future research on GSCM should consider these results, form a more comprehensive understanding of supply chain process ambidexterity, and expand the applicability of the model in multiple scenarios.

## Data availability statement

The raw data supporting the conclusions of this article will be made available by the authors, without undue reservation.

## Ethics statement

Ethical review and approval were not required for the study on human participants in accordance with the local legislation and institutional requirements. Written informed consent for participation was not required for this study in accordance with national legislation and institutional requirements. Written informed consent was obtained from the individual(s) for the publication of any potentially identifiable images or data included in this article.

## Author contributions

TL was responsible for idea generation, manuscript writing, and revision. YG was responsible for hypothesis development and data analysis. HL was responsible for data collection and initial manuscript writing. All authors contributed to the article and approved the submitted version.

## Funding

This study was supported by the Natural Science Foundation of Shandong Province of China (No. ZR2021QG007) and the Humanities and Social Science Fund of the Ministry of Education of China (No. 19YJC630118).

## Conflict of interest

The authors declare that the research was conducted in the absence of any commercial or financial relationships that could be construed as a potential conflict of interest.

## Publisher’s note

All claims expressed in this article are solely those of the authors and do not necessarily represent those of their affiliated organizations, or those of the publisher, the editors and the reviewers. Any product that may be evaluated in this article, or claim that may be made by its manufacturer, is not guaranteed or endorsed by the publisher.
